# 氟马替尼与伊马替尼治疗初发慢性髓性白血病慢性期患者的有效性及严重血液学不良反应发生率比较

**DOI:** 10.3760/cma.j.issn.0253-2727.2023.09.005

**Published:** 2023-09

**Authors:** 小帅 张, 兵城 刘, 新 杜, 龑莉 张, 娜 许, 晓力 刘, 纬明 黎, 海 林, 蓉 梁, 春燕 陈, 健 黄, 云帆 杨, 焕玲 朱, 崚 潘, 晓冬 王, 国辉 李, 卓刚 刘, 延清 张, 振芳 刘, 建达 胡, 春水 刘, 菲 李, 威 杨, 力 孟, 艳秋 韩, 丽娥 林, 震宇 赵, 传清 涂, 彩凤 郑, 炎亮 白, 泽平 周, 苏宁 陈, 惠英 仇, 莉洁 杨, 秀丽 孙, 慧 孙, 励 周, 泽林 刘, 淡瑜 王, 健欣 郭, 丽萍 庞, 庆曙 曾, 晓慧 索, 伟华 张, 媛君 郑, 倩 江

**Affiliations:** 1 北京大学人民医院、北京大学血液病研究所、国家血液系统疾病临床医学研究中心，北京 100044 Peking University People's Hospital, Peking University Institute of Hematology, National Clinical Research Center for Hematologic Disease, Beijing Key Laboratory of Hematopoietic Stem Cell Transplantation, Beijing 100044, China; 2 中国医学科学院血液病医院（中国医学科学院血液学研究所），天津 300020 National Clinical Research Center for Blood Diseases, Institute of Hematology & Blood Diseases Hospital, Chinese Academy of Medical Sciences & Peking Union Medical College, Tianjin 300020, China; 3 深圳市第二人民医院，深圳 518035 The Second People's Hospital of Shenzhen, Shenzhen 518035, China; 4 郑州大学附属肿瘤医院、河南省肿瘤医院、河南省血液病研究所，郑州 450008 Henan Cancer Hospital, The Affiliated Cancer Hospital of Zhengzhou University, Zhengzhou 450008, China; 5 南方医科大学南方医院，广州 510515 Nanfang Hospital, Southern Medical University,Guangzhou 510515, China; 6 华中科技大学同济医学院附属协和医院，武汉 430022 Union Hospital, Tongji Medical College, Huazhong University of Science and Technology, Wuhan 430022, China; 7 吉林大学第一医院，长春 130021 First Hospital of Jilin University, Changchun 130021, China; 8 空军军医大学西京医院，西安 710032 Xijing Hospital, Airforce Military Medical University, Xi'an 710032, China; 9 山东大学齐鲁医院，济南 250012 Qilu Hospital of Shandong University, Jinan 250012, China; 10 浙江大学医学院附属第四医院，杭州 322000 The Fourth Affiliated Hospital of Zhejiang University, Hangzhou 322000, China; 11 四川大学华西医院，成都 610041 Institute of Hematology, West China Hospital, Sichuan University, Chengdu 610041, China; 12 四川省人民医院，成都 610072 Sichuan Academy of Medical Sciences Sichuan Provincial People's Hospital, Chengdu 610072, China; 13 空军军医大学第二附属医院，西安 710038 Xi'an International Medical Center Hospital, Xi'an 710038, China; 14 中国医科大学附属盛京医院，沈阳 110020 Shengjing Hospital of China Medical University, Shenyang 110020, China; 15 哈尔滨医科大学附属第二医院，哈尔滨 150086 The Second Affiliated Hospital of Harbin Medical University, Harbin 150086, China; 16 广西医科大学第一附属医院，南宁 530021 The First Affiliated Hospital of Guangxi Medical University, Nanning 530021, China; 17 福建医科大学附属协和医院，福建省血液病研究所，福建省血液病学重点实验室，福州 350001 Fujian Medical University Union Hospital, Fuzhou 350001, China; 18 南昌大学第一附属医院，南昌大学淋巴肿瘤疾病研究所，南昌 330006 The First Affiliated Hospital of Nanchang University, Nanchang 330006, China; 19 中国医科大学附属盛京医院，沈阳 110020 Shengjing Hospital of China Medical University, Shenyang 110020, China; 20 华中科技大学同济医学院附属同济医院，武汉 430030 Tongji Hospital of Tongji Medical College, Tongji Medical College of Huazhong University of Science and Technology, Wuhan 430030, China; 21 内蒙古医科大学附属医院，呼和浩特 010050 The Affiliated Hospital of Inner Mongolia Medical University, Hohhot 010050, China; 22 海南省人民医院，海口 570311 Hainan General Hospital, Haikou 570311, China; 23 深圳市宝安区人民医院，深圳 518101 Shenzhen Baoan Hospital, Shenzhen University Second Affiliated Hospital, Shenzhen 518101, China; 24 河南省人民医院，郑州 450003 Henan Provincial People's Hospital, Zhengzhou University People's Hospital, Zhengzhou 450003, China; 25 昆明医科大学第二附属医院，昆明 650106 The Second Hospital Affiliated to Kunming Medical University, Kunming 650106, China; 26 苏州大学附属第一医院，江苏省血液研究所，国家血液系统疾病临床医学研究中心，国家卫生健康委员会血栓与止血重点实验室，血液学协同创新中心，苏州 215006 National Clinical Research Center for Hematologic Diseases, Jiangsu Institute of Hematology, The First Affiliated Hospital of Soochow University, Institute of Blood and Marrow Transplantation of Soochow University, Suzhou 215006, China; 27 西安国际医学中心医院，西安 710117 Xi'an International Medical Center Hospital, Xi'an 710117, China; 28 大连医科大学附属第一医院，大连 116011 The First Affiliated Hospital of Dalian Medical University, Dalian 116011, China; 29 郑州大学第一附属医院血液科，郑州 450000 The First Affiliated Hospital of Zhengzhou University, Zhengzhou 450000, China; 30 上海交通大学附属瑞金医院，上海 200025 Shanghai Institute of Hematology, State Key Laboratory of Medical Genomics, National Research Center for Translational Medicine at Shanghai, Ruijin Hospital Affiliated to Shanghai Jiao Tong University School of Medicine, Shanghai 200025, China; 31 华中科技大学协和深圳医院（南山医院），深圳 518000 Huazhong University of Science and Technology Union Shenzhen Hospital, Nanshan Hospital, Shenzhen 518000, China; 32 福建医科大学附属第二医院，泉州 362000 The Second Affiliated Hospital of Fujian Medical University, Quanzhou 362000, China; 33 北京大学深圳医院，深圳 516473 Peking University Shenzhen Hospital, Shenzhen 516473, China; 34 安徽医科大学第一附属医院，合肥 230022 The First Affiliated Hospital of Anhui Medical University, Hefei 230022, China; 35 邯郸市中心医院，邯郸 057150 Handan Central Hospital, Handan 057150, China; 36 山西医科大学第一医院，太原 300012 First Hospital of Shangxi Medical University, Taiyuan 300012, China

**Keywords:** 白血病，髓样，慢性, 氟马替尼, 伊马替尼, 疗效, 血液学不良反应, Leukemia, myeloid, chronic (CML), Flumatinib, Imatinib, Efficacy, Severe hematologic adverse events

## Abstract

**目的:**

比较氟马替尼与伊马替尼治疗初发慢性髓性白血病（CML）慢性期患者的治疗反应、结局以及严重血液学不良反应发生率。

**方法:**

回顾性收集来自国内76个中心自2006年1月至2022年11月期间确诊、年龄≥18岁、确诊后6个月内接受氟马替尼或伊马替尼作为一线治疗且临床资料相对完整的CML慢性期病例。通过倾向性评分匹配（PSM）减少一线酪氨酸激酶抑制剂（TKI）药物选择偏倚，比较两种TKI的治疗反应及结局。

**结果:**

研究最终纳入4 833例接受伊马替尼（4 380例）和氟马替尼（453例）作为一线治疗的成人CML慢性期患者。伊马替尼治疗组中位随访54（*IQR*：31～85）个月，7年完全细胞遗传学反应（CCyR）、主要分子学反应（MMR）、分子学反应4（MR^4^）和分子学反应4.5（MR^4.5^）累积获得率分别为95.2％、88.4％、78.3％和63.0％。7年无治疗失败生存（FFS）率、无进展生存（PFS）率和总生存（OS）率分别为71.8％、93.0％和96.9％。氟马替尼治疗组中位随访18（*IQR*：13～25）个月，2年CCyR、MMR、MR^4^和MR^4.5^累积获得率分别为95.4％、86.5％、58.4％和46.6％。2年FFS率、PFS率和OS率分别为80.1％、95.0％和99.5％。PSM分析显示，氟马替尼治疗组患者CCyR、MMR、MR^4^和MR^4.5^累积获得率及FFS率均显著高于伊马替尼治疗组患者（*P*值均<0.001），但两组患者PFS率（*P*＝0.230）和OS率（*P*＝0.268）差异无统计学意义。两组患者Ⅲ级及以上血液学不良反应的发生率相似（氟马替尼对伊马替尼：10.6％对8.0％）。

**结论:**

氟马替尼治疗初发CML慢性期患者的治疗反应获得率及FFS率均高于伊马替尼，且严重血液学不良反应发生率相似。

## Abstract (en)

**Objective:**

To analyze and compare therapy responses, outcomes, and incidence of severe hematologic adverse events of flumatinib and imatinib in patients newly diagnosed with chronic phase chronic myeloid leukemia (CML).

**Methods:**

Data of patients with chronic phase CML diagnosed between January 2006 and November 2022 from 76 centers, aged ≥18 years, and received initial flumatinib or imatinib therapy within 6 months after diagnosis in China were retrospectively interrogated. Propensity score matching (PSM) analysis was performed to reduce the bias of the initial TKI selection, and the therapy responses and outcomes of patients receiving initial flumatinib or imatinib therapy were compared.

**Results:**

A total of 4 833 adult patients with CML receiving initial imatinib (*n*＝4 380) or flumatinib (*n*＝453) therapy were included in the study. In the imatinib cohort, the median follow-up time was 54 [interquartile range (IQR), 31–85] months, and the 7-year cumulative incidences of CCyR, MMR, MR^4^, and MR^4.5^ were 95.2％, 88.4％, 78.3％, and 63.0％, respectively. The 7-year FFS, PFS, and OS rates were 71.8％, 93.0％, and 96.9％, respectively. With the median follow-up of 18 (IQR, 13–25) months in the flumatinib cohort, the 2-year cumulative incidences of CCyR, MMR, MR^4^, and MR^4.5^ were 95.4％, 86.5％, 58.4％, and 46.6％, respectively. The 2-year FFS, PFS, and OS rates were 80.1％, 95.0％, and 99.5％, respectively. The PSM analysis indicated that patients receiving initial flumatinib therapy had significantly higher cumulative incidences of CCyR, MMR, MR^4^, and MR^4.5^ and higher probabilities of FFS than those receiving the initial imatinib therapy (all *P*<0.001), whereas the PFS (*P*＝0.230) and OS (*P*＝0.268) were comparable between the two cohorts. The incidence of severe hematologic adverse events (grade≥Ⅲ) was comparable in the two cohorts.

**Conclusion:**

Patients receiving initial flumatinib therapy had higher cumulative incidences of therapy responses and higher probability of FFS than those receiving initial imatinib therapy, whereas the incidence of severe hematologic adverse events was comparable between the two cohorts.

酪氨酸激酶抑制剂（TKI）的问世，显著延长了慢性髓性白血病（CML）患者的生存期，并提高了患者的生活质量[1]–[3]。伊马替尼作为一线药物治疗初发CML慢性期患者，10年生存率可达80％～90％[4]。而随后几年出现的第二代TKI（尼洛替尼、达沙替尼）可使患者获得更快、更深的细胞遗传学及分子学反应，也为伊马替尼耐药和（或）不耐受患者提供了更多选择[5]–[7]。氟马替尼作为中国自主研发的第二代TKI，于2019年11月在我国正式上市，并被用于CML一线治疗。临床试验结果显示，与一线伊马替尼治疗相比，氟马替尼可使更高比例患者更快获得细胞遗传学及分子学反应，12个月无进展生存（PFS）率更高，且血液学不良反应发生率更低[8]。然而，该临床试验随访时间较短，且目前尚缺乏真实世界下氟马替尼与伊马替尼一线治疗CML的疗效和安全性比较的研究。因此，我们收集了来自中国76家中心一线氟马替尼和伊马替尼治疗的4 833例初发CML患者数据，进一步分析比较两种TKI治疗CML的疗效以及严重血液学不良反应发生率。

## 病例与方法

一、病例

回顾性收集来自国内76个中心自2006年1月至2022年11月期间确诊、年龄≥18岁、确诊后6个月内接受伊马替尼或氟马替尼作为一线治疗且临床资料相对完整的CML慢性期病例资料。由于氟马替尼2019年11月上市，因此纳入本研究的一线氟马替尼治疗的患者均为2019年11月至2022年11月期间确诊的患者。收集初诊患者性别、年龄、全血细胞计数、外周血原始细胞及嗜碱性粒细胞比例、Sokal和ELTS评分、初诊染色体核型、合并症、一线TKI种类和剂量、治疗期间出现的Ⅲ级及以上血液学不良反应、治疗反应和结局等。

二、诊断分期

参照欧洲白血病网（ELN）推荐的CML诊断和分期标准[2],[9]–[11]。

三、治疗

纳入研究的患者初始均采用标准剂量（伊马替尼400 mg或氟马替尼600 mg每日1次），治疗中根据治疗反应和（或）不良反应等调整TKI剂量或类型。

四、监测

参照2006、2009、2013及2020年ELN推荐进行治疗反应监测[2],[9]–[11]。

1. 血液学：每1～2周进行血细胞计数和分类，直至达到完全血液学反应（CHR）后，每3个月进行1次。

2. 细胞遗传学：每3个月采用染色体G显带法进行骨髓细胞遗传学分析，达到完全细胞遗传学反应（CCyR）后，可暂停检测；当患者出现（可疑）治疗失败或疾病进展时，重启骨髓细胞遗传学检测。

3. 分子学：每3个月通过实时定量聚合酶链反应（RQ-PCR）检测外周血BCR∷ABL转录本水平，以ABL为内参基因，ABL拷贝数>32 000。直至获得主要分子学反应（MMR）后，每3～6个月进行1次；当出现警告或治疗失败时，通过Sanger测序检测ABL激酶区突变情况。

五、评估指标

1. 血液学不良反应：根据CTCAE 4.03版本进行分级[12]。因Ⅲ级及以上血液学不良反应可导致患者TKI剂量调整、暂时停药或更换TKI治疗，故本研究只关注Ⅲ级及以上血液学不良反应事件。

2. 治疗反应：根据ELN推荐，定义CHR、CCyR、MMR、分子学反应4（MR^4^）和分子学反应4.5（MR^4.5^）[2],[9]–[11]。

3. 结局：无治疗失败生存（FFS）期定义为从开始TKI治疗至首次治疗失败或末次随访的时间；PFS期定义为从开始TKI治疗至首次进展到加速/急变期、死亡或末次随访的时间；总生存（OS）期定义为从开始TKI治疗至死亡或末次随访的时间。所有结局观察均删失至造血干细胞移植。

六、随访

采用门诊或电话联系的方式进行随访，末次随访时间为2023年2月。

七、统计学处理

对于患者基本特征包括社会人口学资料及临床特征采用描述性统计学分析，计量资料采用*M*（范围）或*M*［四分位间距（*IQR*）］表示，计数资料采用例数（％）表示。对数据进行组间比较时，连续变量正态分布数据采用*t*检验，非正态分布数据采用Mann-Whitney *U*非参数检验，分类变量进行卡方检验或Fisher精确概率法。对于累积治疗反应获得率采用竞争风险模型分析，竞争风险事件为移植或死亡，并应用Fine-Grey检验进行组间比较；对于结局采用Kaplan-Meier生存曲线分析，并应用Log-rank检验进行组间比较。为更好地比较氟马替尼和伊马替尼一线治疗CML患者的治疗反应及结局，减少两组患者基线特征偏倚，同时考虑到氟马替尼上市时间晚于伊马替尼的情况，可能造成两组患者TKI药物可及性等差异，因此，我们仅纳入2019年11月后诊断的、接受一线伊马替尼或氟马替尼治疗的初发CML患者，通过倾向性评分匹配（PSM）减少一线TKI药物选择偏倚，使用年龄、性别、全血细胞计数、外周血原始细胞及嗜碱性粒细胞百分比、ELTS评分、附加染色体异常、合并症等变量为协变量，以1∶2比例匹配，卡嵌值设置为0.2。*P*<0.05为差异有统计学意义。采用SPSS 22.0、R 4.0.2及GraphPad Prism 8软件进行统计分析、绘图。

## 结果

1. 患者特征：2006年1月至2022年11月，共收集到来自76个中心的5 674例≥18岁、接受一线伊马替尼或氟马替尼治疗的初发CML连续性病例资料，排除各中心提供的重复病例资料339例，进展期（包括加速期、急变期）及疾病分期不详119例，关键临床信息缺失（包括初诊时全血细胞计数及分类、初诊染色体核型等）205例、不规律随访或失访178例，最终本研究纳入具有较完整社会人口学信息及临床特征资料的4 833例接受伊马替尼（4 380例）或氟马替尼（453例）作为一线治疗的成人CML慢性期患者。中位年龄43（18～86）岁，男性2 895例（60.0％）。ELTS评分低危2 878例（59.5％）、中危1 039例（21.4％）、高危298例（6.1％），其余618例（12.7％）患者ELTS危险度分组不详。1 143例（23.6％）例患者初诊时伴有合并症。59例（1.2％）初诊时携带高危附加染色体异常。

与一线伊马替尼治疗人群相比，一线氟马替尼治疗人群年龄更大（*P*＝0.009），Sokal和ELTS评分中、高危患者比例更高（*P*＝0.006和0.011），初诊时伴有合并症的患者比例更高（*P*＝0.002）。两组间性别、初诊时全血细胞计数、外周血原始细胞及嗜碱性粒细胞比例、脾脏肋缘下厘米数、初诊时携带高危附加染色体异常比例、诊断至开始TKI治疗时间差异均无统计学意义（表1）。

**表1:** 4 833例初发慢性髓性白血病慢性期患者特征

变量	患者总体（4 833例）	一线伊马替尼组（4 380例）	一线氟马替尼组（453例）	统计量（*χ^2^/t*）	*P*值
年龄［岁，*M*（范围）］	43（18~86）	42（18~86）	43（18~80）	−2.604	0.009
男性［例（％）］	2 895（60.0）	2 619（59.7）	276（60.9）	0.219	0.640
WBC［×10^9^/L，*M*（范围）］	119.8（1.7~874.4）	118.8（1.7~874.4）	135.2（3.8~791.7）	−1.380	0.168
HGB［g/L，*M*（范围）］	113.0（18.0~340.0）	113.6（18.0~340.0）	112.0（45.0~186.0）	0.780	0.435
PLT［×10^9^/L，*M*（范围）］	418（36~4 511）	415（36~4 511）	445（72~2 500）	−1.501	0.133
外周血原始细胞［％，*M*（范围）］	1.0（0~14.0）	1.0（0~14.0）	0.5（0~14.0）	−1.795	0.073
外周血嗜碱性粒细胞［％，*M*（范围）］	4.0（0~19.0）	4.0（0~19.0）	4.0（0~19.0）	−0.659	0.510
脾脏［cm，*M*（范围）］^a^	3（0~32）	3（0~32）	3（0~23）	0.643	0.521
Sokal危险度［例（％）］				12.441	0.006
低危	1 974（40.8）	1 795（41.0）	179（39.5）		
中危	1 491（30.9）	1 351（30.8）	140（30.9）		
高危	750（15.5）	658（15.0）	92（20.3）		
不详	618（12.8）	576（13.2）	42（9.3）		
ELTS危险度［例（％）］				11.156	0.011
低危	2 878（59.5）	2 617（59.7）	261（57.6）		
中危	1 039（21.4）	926（21.1）	113（24.9）		
高危	298（6.1）	261（5.9）	37（8.1）		
不详	618（12.7）	576（13.1）	42（9.2）		
伴有合并症［例（％）］	1 143（23.6）	1 009（23.0）	134（29.5）	9.737	0.002
伴有高危附加染色体异常［例（％）］	59（1.2）	51（1.1）	8（1.7）	1.356	0.252
诊断到开始TKI治疗时间［月，*M*（范围）］	0.3（0~5.6）	0.3（0~5.6）	0.2（0~4.8）	0.124	0.682

注 ^a^肋缘下厘米数；ELTS：EUTOS长期生存评分；TKI：酪氨酸激酶抑制剂

由于氟马替尼问世晚于伊马替尼，氟马替尼治疗组患者中位随访时间显著短于伊马替尼治疗组［18（*IQR*：13～25）个月对54（*IQR*：31～85）个月；*P*<0.001］。截至末次随访，3 452例（78.8％）患者仍在接受初始的伊马替尼治疗，401例（88.5％）患者仍在接受初始的氟马替尼治疗。

2．伊马替尼治疗组治疗反应及结局：在伊马替尼治疗组患者中，4 197例（95.9％）可评估CCyR状态及获得时间，4 225例（96.5％）、4 154例（94.9％）可分别评估MMR、深层分子学反应（包括MR^4^和MR^4.5^）状态及获得时间；4 135例（94.4％）、4 190例（95.7％）可分别评估治疗失败、疾病进展状态及时间；所有患者均可评估生存状态及时间。在上述可评估的患者中，中位随访54（*IQR*：31～85）个月，3 773例（90.0％）、3 379例（80.0％）、2 559例（61.6％）、2 079例（50.0％）分别获得CCyR、MMR、MR^4^和MR^4.5^。随访期内，1 040例（25.2％）患者至少经历1次治疗失败；219例（5.2％）疾病进展至加速期（112例，2.7％）或急变期（107例，2.5％）；99例（2.2％）死于疾病进展（82例，1.9％）或非CML相关原因（17例，0.3％）。

在至少经历1次治疗失败的1 040例患者中，752例（72.3％）患者可评估首次治疗失败时的ABL激酶区突变状态。其中，299例（39.8％）患者检出突变。所检出的突变类型根据突变频率排序依次为T315I（42例，12.1％）、Y253H/F（22例，6.4％）、E255K/V（16例，4.6％）和F359V/C/I（13例，4.3％）等（图1A）。

伊马替尼治疗组患者1、2年累积治疗反应获得率见表2。7年CCyR、MMR、MR^4^和MR^4.5^累积获得率分别为95.2％（95％*CI* 93.1％～97.3％）、88.4％（95％*CI* 85.9％～90.9％）、78.3％（95％*CI* 73.6％～82.9％）、63.0％（95％*CI* 55.1％～70.8％）。7年FFS、PFS和OS率分别为71.8％（95％*CI* 70.2％～73.4％）、93.0％（95％*CI* 85.9％～99.2％）、96.9％（95％*CI* 90.9％～100％）。

**图1:**
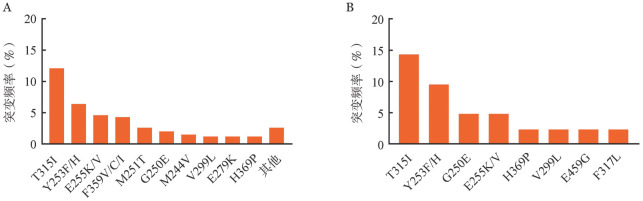
伊马替尼（A）和氟马替尼（B）治疗失败时ABL突变检出频率

**表2:** 伊马替尼和氟马替尼一线治疗4 833例初发慢性髓性白血病慢性期患者累积治疗反应获得率及生存率（％）

治疗反应或生存	伊马替尼组			氟马替尼组	
	1年	2年	7年	1年	2年
CCyR	82.4（81.1~83.7）	89.5（87.3~91.6）	95.2（93.1~97.3）	92.6（89.4~95.8）	95.4（92.3~98.5）
MMR	50.7（48.3~53.1）	72.3（66.5~78.1）	88.4（85.9~90.9）	67.8（62.0~73.5）	86.5（77.7~95.3）
MR^4^	22.7（18.0~27.4）	42.6（37.4~47.8）	78.3（73.6~82.9）	36.5（27.1~45.9）	58.4（51.0~65.8）
MR^4.5^	9.6（4.6~14.5）	25.0（18.6~31.4）	63.0（55.1~70.8）	21.7（10.6~32.8）	46.6（37.1~55.1）
FFS	82.9（80.4~85.4）	78.7（75.1~82.3）	71.8（70.2~73.4）	83.9（80.0~87.8）	80.1（75.0~85.2）
PFS	97.3（95.0~99.6）	96.2（92.9~99.5）	93.0（85.9~99.2）	96.6（94.4~98.8）	95.0（90.8~99.2）
OS	99.7（98.5~100）	98.9（97.1~100）	96.9（90.9~100）	99.5（97.9~100）	99.5（96.3~100）

注 由于氟马替尼上市并被用于慢性髓性白血病一线治疗时间（2019年11月）晚于伊马替尼，故接受一线氟马替尼治疗的患者总体随访时间显著短于伊马替尼，仅伊马替尼组患者治疗反应及生存率可评估至7年。CCyR：完全细胞遗传学反应；MMR：主要分子学反应；MR^4^：分子学反应4；MR^4.5^：分子学反应4.5；FFS：无治疗失败生存；PFS：无进展生存；OS：总生存

3. 氟马替尼治疗组治疗反应及结局：在氟马替尼治疗组患者中，412例（90.9％）可评估CCyR状态及获得时间，407例（89.8％）、390例（86.1％）可分别评估MMR、深层分子学反应（包括MR^4^和MR^4.5^）状态及获得时间；447例（98.7％）可评估治疗失败；所有患者均可评估疾病进展、生存状态及时间。在上述可评估的患者中，中位随访18（*IQR*：13～25）个月，377例（91.5％）、275例（67.6％）、177例（45.4％）、131例（33.6％）获得CCyR、MMR、MR^4^和MR^4.5^。随访期内，51例（11.4％）患者至少经历1次治疗失败；12例（2.6％）疾病进展至加速期（7例，1.5％）或急变期（5例，1.1％）；仅2例（0.4％）死亡，均死于疾病进展。

在至少经历1次治疗失败的51例患者中，42例（82.4％）患者可评估首次治疗失败时的ABL激酶区突变状态。其中，18例（42.9％）患者检出突变。所检出的突变类型根据突变频率排序依次为T315I（6例，14.3％）、Y253F/H（4例，9.5％）和G250E（2例，4.8％）等突变（图1B）。

氟马替尼治疗组患者1年累积治疗反应获得率见表2。2年CCyR、MMR、MR^4^和MR^4.5^累积获得率分别为95.4％（95％*CI* 92.3％～98.5％）、86.5％（95％*CI* 77.7％～95.3％）、58.4％（95％*CI* 51.0％～65.8％）、46.6％（95％*CI* 37.1％～55.1％）。2年FFS、PFS和OS率分别为80.1％（95％*CI* 75.0％～85.2％）、95.0％（95％*CI* 90.8％～99.2％）、99.5％（95％*CI* 96.3％～100％）。

4. PSM分析比较伊马替尼和氟马替尼治疗组治疗反应及结局：纳入2019年11月以后确诊的、接受一线伊马替尼（1 439例）或氟马替尼治疗（453例）的初发CML患者，共1 892例。使用年龄、性别、全血细胞计数、外周血原始细胞及嗜碱性粒细胞百分比、Sokal和ELTS评分、附加染色体异常、合并症等变量作为匹配协变量，以1∶2比例匹配后，最终，共纳入1 212例CML患者，其中一线伊马替尼治疗808例，一线氟马替尼治疗404例。匹配后两组患者基线特征差异均无统计学意义。

对匹配后患者的治疗反应及结局比较发现，氟马替尼治疗组患者CCyR、MMR、MR^4^和MR^4.5^累积获得率及FFS率均显著高于伊马替尼治疗组患者（*P*值均<0.001），但两组患者PFS（*P*＝0.230）和OS（*P*＝0.268）率差异并无统计学意义（图2）。随后，我们在不同ELTS危险度分层患者中分别比较了伊马替尼和氟马替尼的治疗反应和结局。在ELTS低、中危患者中，氟马替尼治疗组CCyR、MMR、MR^4^和MR^4.5^累积获得率（*P*值均<0.001）及FFS率（*P*＝0.002和0.033）均显著高于伊马替尼治疗组，但两组患者PFS率（*P*＝0.342和0.589）和OS率（*P*＝0.104和0.502）差异并无统计学意义。在ELTS高危患者中，尽管氟马替尼治疗组CCyR、MMR和MR^4^累积获得率显著高于伊马替尼治疗组（*P*值均<0.001），两组患者MR^4.5^累积获得率（*P*＝0.216）、FFS率（*P*＝0.183）、PFS率（*P*＝0.108）和OS率（*P*＝0.522）差异并无统计学意义。

**图2:**
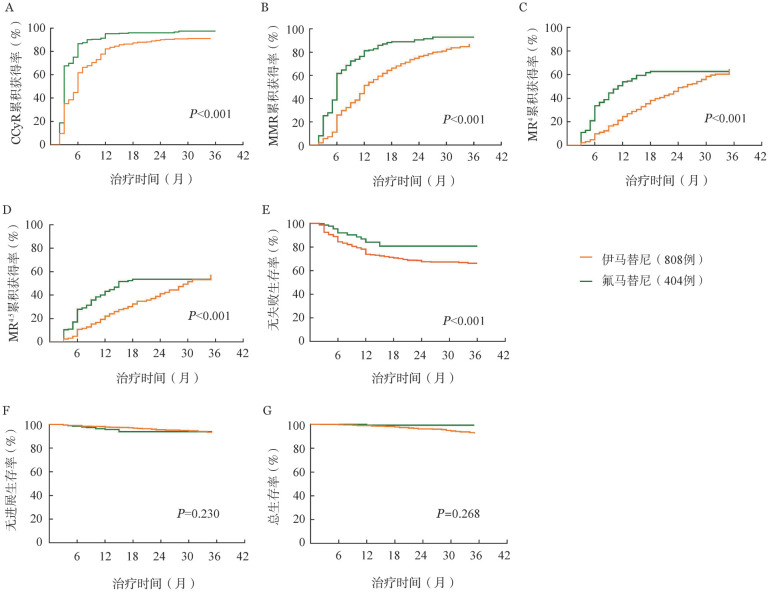
比较倾向性评分匹配后伊马替尼和氟马替尼一线治疗慢性髓性白血病慢性期患者的治疗反应及结局（A～D：治疗反应；E～G：结局）

5. 伊马替尼和氟马替尼严重血液学不良反应发生率的比较：在4 380例伊马替尼治疗组患者中，2 796例（63.8％）可评估Ⅲ级及以上血液学不良反应发生情况。伊马替尼治疗过程中，共223例（8.0％）出现Ⅲ级及以上血液学不良反应，包括血小板减少（131例，4.7％）、白细胞减少（95例，3.4％）和（或）贫血（62例，2.2％）。在453例氟马替尼治疗组患者中，所有患者均可评估Ⅲ级及以上血液学不良反应发生情况。氟马替尼治疗过程中，共48例（10.6％）出现Ⅲ级及以上血液学不良反应，包括血小板减少（28例，6.2％）、白细胞减少（14例，3.1％）和（或）贫血（8例，1.8％）。

比较发现，尽管氟马替尼治疗组患者Ⅲ级及以上血液学不良反应发生率略高于一线伊马替尼治疗组患者（10.6％ 对8.0％, *P*＝0.061），但Ⅲ级及以上血小板减少（*P*＝0.171）、白细胞减少（*P*＝0.736）和（或）贫血（*P*＝0.539）的发生率与伊马替尼治疗组比较差异并无统计学意义。

## 讨论

本研究通过回顾性分析来自全国76个中心的4 833例初发CML慢性期病例，分析比较了真实世界下伊马替尼和氟马替尼一线治疗CML的治疗反应、结局和Ⅲ级及以上血液学不良反应发生率。研究结果显示，氟马替尼治疗组患者细胞遗传学、分子学反应累积获得率和FFS率均显著高于伊马替尼治疗组患者，尤其是在ELTS评分低、中危患者中，且伊马替尼和氟马替尼治疗期间Ⅲ级及以上血液学不良反应发生率相似。

FESTnd Ⅲ期临床试验数据显示，氟马替尼一线治疗CML慢性期患者6、9和12个月MMR、MR^4^和MR^4.5^率均显著高于伊马替尼，12个月PFS率更高，且Sokal评分低、中危组患者接受一线氟马替尼治疗CCyR、MMR获得率高于伊马替尼[8]。徐娟等[13]也发现，一线接受氟马替尼治疗的CML患者3、6个月早期分子学反应以及6、12个月MMR获得率显著高于伊马替尼治疗组患者。与我们的回顾性研究结果一致的是，氟马替尼治疗组患者CCyR、MMR、MR^4^和MR^4.5^累积获得率均显著高于伊马替尼治疗组患者。此外，我们在回顾性研究中还比较了FFS，分析发现氟马替尼组患者FFS率显著高于伊马替尼组。但我们并未发现两组间PFS和OS率的差异。作为TKI时代提出的ELTS危险度评分，有研究显示，其相较于Sokal评分系统具有更好的治疗反应和结局预测能力，可用于指导一线TKI治疗选择[14]–[15]。本研究结果显示，一线接受氟马替尼治疗的ELTS低、中危患者CCyR、MMR、MR^4^和MR^4.5^累积获得率以及FFS率均显著高于伊马替尼治疗组患者。而在ELTS高危组患者中，一线氟马替尼治疗仅使患者获益于更高的CCyR、MMR和MR^4^累积获得率，而MR^4.5^、FFS、PFS和OS相似。同时我们也对纳入本研究可评估Ⅲ级及以上血液学不良反应发生情况的患者分析显示，氟马替尼治疗期间严重血液学不良反应包括血小板减少、白细胞减少、贫血发生率与伊马替尼治疗组相似，但均低于既往在FESTnd Ⅲ临床试验中所报道的比例[8]。其可能的原因是，纳入本研究的氟马替尼人群Sokal和ELTS评分中高危人群总体比例低于FESTnd Ⅲ研究入组人群。既往研究显示，Sokal中高危患者TKI治疗期间Ⅲ级及以上血液学不良反应发生率更高[16]–[17]。

既往研究报道，CML患者出现伊马替尼治疗失败时，15％～55％的患者可检出ABL突变，包括T315I、Y253F/H、F359I/V/C、E255K/V、M244V突变等[18]–[19]。而当患者一线接受氟马替尼治疗失败时的ABL突变检出情况尚未见报道。本研究结果显示，39.8％的伊马替尼治疗失败患者检出ABL突变，其中T315I突变发生率最高，其次为Y253H/F、E255K/V和F359V/C/I等突变，与既往报道结果相似[18]–[19]。一线接受氟马替尼治疗失败的患者中，42.9％的患者检出ABL突变，其中T315I突变发生率最高，其次为E255K/V和G250E等突变。这是首次报道氟马替尼治疗失败时的ABL突变状态。但由于本研究纳入的氟马替尼治疗的患者例数有限，且随访时间较短，未来还需要扩大样本量的研究予以进一步证实。

本研究有以下局限性：①为回顾性、多中心研究，纳入研究的患者尤其是接受伊马替尼治疗的患者数量较大，无法对其治疗期间的服药、监测依从性进行严格把控，仅能呈现真实世界下的数据；②纳入研究的氟马替尼治疗组患者数量有限，相较于伊马替尼治疗组随访时间也显著更短，未来需要更大样本量、更长随访时间的研究，予以进一步探索和验证；③由于本研究中部分来自其他中心的患者监测频率不规范和（或）检测技术敏感性的限制，以及原始数据保留情况较差，导致部分患者无法得知其获得细胞遗传学、分子学反应的具体时间，部分患者治疗失败的准确时间及失败时ABL突变状态也无法获得；④本研究仅收集并分析了患者接受伊马替尼或氟马替尼治疗期间出现严重（Ⅲ级及以上）血液学不良反应的情况，并未对轻中度（Ⅰ～Ⅱ级）血液学不良反应及其他非血液学不良反应发生情况进行收集和分析。这些因素对研究结果可能产生一定影响。

总之，我们通过分析来自国内76个中心的4 833例初发CML患者数据发现，一线氟马替尼治疗初发CML慢性期患者的细胞遗传学、分子学反应获得率及FFS率高于伊马替尼，且严重血液学不良反应发生率相似。氟马替尼的问世为CML患者提供了一个安全有效的治疗手段，其长期疗效和安全性有待大宗病例的临床研究进一步证实。
